# Preparation and Characterization of Nanocomposite Polymer Membranes Containing Functionalized SnO_2_ Additives

**DOI:** 10.3390/membranes4010123

**Published:** 2014-03-05

**Authors:** Roberto Scipioni, Delia Gazzoli, Francesca Teocoli, Oriele Palumbo, Annalisa Paolone, Neluta Ibris, Sergio Brutti, Maria Assunta Navarra

**Affiliations:** 1 Dipartimento di Chimica, Sapienza Università di Roma, Piazzale Aldo Moro 5, 00185 Roma, Italy; E-Mails: roberto.scipioni@live.it (R.S.); delia.gazzoli@uniroma1.it (D.G.); 2 Centro di Ricerca Hydro-Eco, Sapienza Università di Roma, Piazzale Aldo Moro 5, 00185 Roma, Italy; E-Mail: fteocoli@gmail.com; 3 Istituto dei Sistemi Complessi-Consiglio Nazionale delle Ricerche–ISC-CNR U.O.S. Sapienza, Piazzale A. Moro 5, 00185 Roma, Italy; E-Mails: oriele.palumbo@roma1.infn.it (O.P.); annalisa.paolone@roma1.infn.it (A.P.); sergio.brutti@unibas.it (S.B.); 4 Dipartimento di Scienze, Università della Basilicata, Viale dell’Ateneo Lucano 10, 85100 Potenza, Italy; E-Mail: neluta.ibris@unibas.it

**Keywords:** functionalized metal oxides, nanocomposite polymer membranes, morphological, structural and spectroscopic characterizations, thermal and mechanical properties

## Abstract

In the research of new nanocomposite proton-conducting membranes, SnO_2_ ceramic powders with surface functionalization have been synthesized and adopted as additives in Nafion-based polymer systems. Different synthetic routes have been explored to obtain suitable, nanometer-sized sulphated tin oxide particles. Structural and morphological characteristics, as well as surface and bulk properties of the obtained oxide powders, have been determined by means of X-ray diffraction (XRD), scanning electron microscopy (SEM), Fourier Transform Infrared (FTIR) and Raman spectroscopies, N_2_ adsorption, and thermal gravimetric analysis (TGA). In addition, dynamic mechanical analysis (DMA), atomic force microscopy (AFM), thermal investigations, water uptake (WU) measurements, and ionic exchange capacity (IEC) tests have been used as characterization tools for the nanocomposite membranes. The nature of the tin oxide precursor, as well as the synthesis procedure, were found to play an important role in determining the morphology and the particle size distribution of the ceramic powder, this affecting the effective functionalization of the oxides. The incorporation of such particles, having sulphate groups on their surface, altered some peculiar properties of the resulting composite membrane, such as water content, thermo-mechanical, and morphological characteristics.

## 1. Introduction

Perfluorosulphonated polymer systems, *i.e.*, Nafion membranes developed by Du Pont, find one of their most challenging application in polymer electrolyte membrane (PEM) fuel cells (FCs), that are a promising technology for both mobile and stationary clean and efficient energy production [1,2,3,4,5,6,7]. Despite undiscussed benefits, such as high proton conductivity, mechanical, and chemical stability, conventional Nafion membranes show properties and performances in FC that limit its mass commercialization. In particular, they show limited duration under cycling voltage, low humidity, and freezing-thawing conditions. Furthermore efficient protons conduction in Nafion membranes is obtained only at high hydration, thus, limiting operating temperatures of PEMFCs to around 80 °C at relative humidity (RH) approaching 100%.

One promising strategy to improve the performance of PEMs is based on the incorporation of nanometer-sized particles of hygroscopic inorganic acids or oxides, which act as a water reservoir into the polymer matrix [8,9]. According to the cluster network model for ionomers, the Nafion microstructure can be visualized as formed by hydrophobic domains (*i.e.*, the fluorinated backbone) and hydrophilic domains, the latter consisting of ionic clusters comprising the sulfonic acid moiety and the absorbed water [10,11]. These clusters are interconnected by a network of short and narrow channels and the size of the clusters is strongly dependent on the water content of the system [12]. Considering the mechanisms of proton transport [13], one may assume that the presence of the inorganic additive, when uniformly dispersed as nano-sized particles in the Nafion matrix, may favorably affect the ionic conductivity. Indeed, the hydration water of the additive can form bridges between shrunken clusters [14] and, in virtue of its acidity, the filler can provide additional pathways for proton hopping from one cluster to another. 

Malhotra and Datta first proposed the use of inorganic solid acids (*i.e.*, heteropolyacids, HPAs) in conventional Nafion, with the objective of serving the dual function of improving water retention, as well as providing additional acidic sites for proton conduction [15].

Similarly to HPAs, sulfated metal oxides have become subjects of intensive studies, being them even more stable than other inorganic solid acid. In virtue of its extraordinary high acidity, sulphated zirconium oxide, SZrO_2_, has attracted interest as fuel cell membrane additive [16,17,18,19,20,21,22,23]. Deeply studied as catalyst for the conversion of organic compounds [24], SZrO_2_ has been rarely investigated as proton conductor [25]. Hara *et al*. found that surface SO_4_ species existed as bidentate complexes strongly bonded with ZrO_2_ in a SZrO_2_ powder calcined at 620 °C. SO_4_-species are electron-withdrawing and, thus, the Lewis acidity of zirconium is strengthened. The Lewis points can easily convert to Brønsted acid points in the presence of water, finally acting as proton donors [26]. Currently, sulphated zirconia is recognized as one of the strongest superacid among all known solids with its Hammett acidity, H_o_ < −16. An extraordinary strong surface acidity, even higher than that of SZrO_2_, has been attributed to sulphated tin oxide, SSnO_2_ [27]. Nevertheless, papers concerning the use of sulphated tin oxide as catalyst have been quite few [27] being it more difficult to obtain especially when large surface area is required [28]. As reported by Arata and co-workers, the main difficulty consists of the preparation of the oxide gels from tin salts, needed for the subsequent treatment with sulfate ions [29]. 

To our knowledge, there is only a recent example on the use of SSnO_2 _in Nafion membranes for direct methanol fuel cells, demonstrating a reduced methanol crossover, improved proton transport, and enhanced membrane/electrode interface stability [30]. 

In this work highly hydrophilic nanosized tin oxide particles without and with sulphated surfaces were obtained by properly adapting well-known synthesis procedures where two different tin oxide precursors were considered [31,32]. The incorporation of the obtained powders into Nafion-based membranes is here described. The goal is to develop a comprehensive description of the properties of these composite materials in order to drive the selection of advanced organic-inorganic hybrid electrolytes for PEM fuel cell applications. 

## 2. Results and Discussion

In the following presentation and discussion of results, all samples, both powders and membranes, are labeled as specified in Section 3.1 and Section 3.2 of the experimental section.

### 2.1. Structure and Morphology of the Bare and Sulphated Tin Oxide Powders

**Figure 1:**
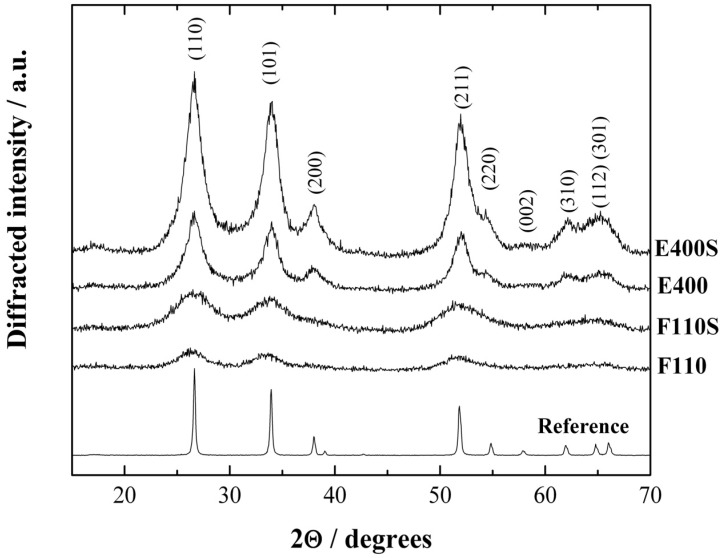
X-ray diffraction (XRD) patterns of the tin oxide samples, bare and sulphated. Reference: SnO_2_ bulk oxide.

The X-ray diffraction (XRD) patterns of the synthesized ceramic samples, both bare and sulphated, are presented in Figure 1.

The XRD patterns of all materials have been indexed in agreement with the tetragonal rutile structure of the bulk SnO_2_ (reference spectra, Sigma Aldrich 99% purity). All the synthesized samples show broad peaks revealing the presence of particles in the nanosized range. The mean crystallite sizes (<d>) have been calculated by the standard Scherrer equation [33] starting from the profile broadening (Δ, full width at half maximum) of the (110), (101), and (211) peaks using the usual formula: <d> = λ/(Δcosθ), being λ = 1.5418 Å the wavelength of the CuKα radiation. The resulting values are 1.7 and 2.8 nm for F110 and F110S samples, and 5.0 and 6.1 nm for E400 and E400S samples, respectively. The various degree of crystallinity reflects the thermal history of the samples. In particular it is to be noted that the sulphated oxides show in both cases (E400S and F110S) larger crystallite sizes compared to the bare pristine oxide (E400 and F110, respectively), this being due to the further annealing step at 500 °C performed during the sulphation procedure. Additionally, the nanosized structure is retained by both E400S and F110S samples. 

**Figure 2:**
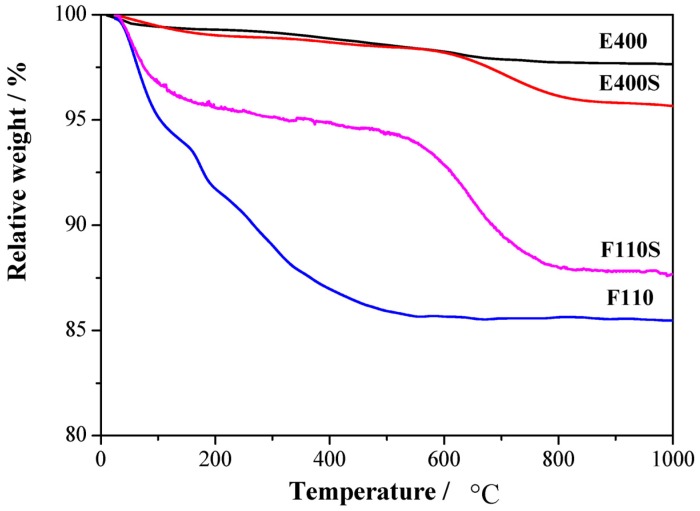
Thermal gravimetric (TG) analysis responses for the four synthesized oxides.

The amount of sulphate groups chemically bonded to the surfaces has been established by thermogravimetric measurements, Figure 2. 

Mass losses are observed in different thermal ranges. The first mass loss below 250 °C is observed in all the four samples and it is related to the removal of physisorbed and chemisorbed water as well as OH groups: the largest mass loss is observed, as expected, for the F110 sample, being it the one treated at relatively low temperature (*i.e.*, 110 °C, see experimental section). A second mass loss is observed between 400 and 500 °C only for the E400 sample and it is likely related to residual organic traces from the organometallic precursor. A similar mass loss in the 400–500 °C range is not evident for the sulphated parent sample E400S, most likely due to the removal of the contaminations during the high temperature treatment involved in the sulphation process. Another mass loss is observed above 600 °C only for the two E400S and F110S sulphated samples. This mass loss is related to the removal of the sulphate groups bonded to the surface of the tin oxide particles [30]. The two samples show a loss of about 3% and 7% in mass for E400S and F110S, respectively. The larger concentration of chemisorbed sulphate groups in the F110S sample compared to the E400S goes in parallel with the smaller crystallite size of the pristine F110 bare oxide before the sulphation process in respect to the E400 one (see XRD results above). Such evidence suggests that the amount of sulphate groups chemisorbed on the SnO_2_ surface increases with the nanometrization of the oxide particles.

**Figure 3:**
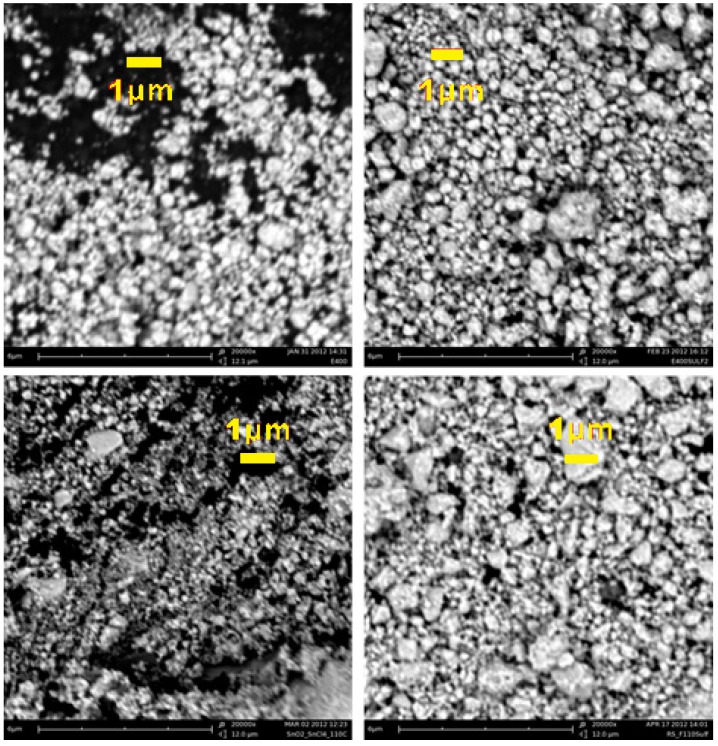
Scanning electron microscopy (SEM) micrographs of the four synthesized oxides. From left to right and from top to bottom: E400, E400S, F110, and F110S, respectively.

The SEM micrographs of the four oxides at 20 k magnifications are reported in Figure 3. 

The morphology in the microscopic range of all the powders is similar: all the samples consist of sub-micrometric particles (secondary particles) with a remarkable dispersion in size. Such large particles are likely formed by the aggregation of the nanometric crystallites estimated by the XRD data. The sulphation of the surfaces does not lead to drastic alterations: the surface-modified materials show only a slight increase in the size of the secondary particles. This picture is also confirmed by the trend of the surface areas, the measured values being 65 ± 2 m^2^ g^−1^ (E400), 52 ± 3 m^2^ g^−1^ (E400S), 160 ± 5 m^2^ g^−1^ (F110) and 100 ± 1 m^2^ g^−1^ (F110S). These values correspond to estimated spherical particle diameters of 13, 17, 5, and 9 nm for the E400, E400S, F110, and F110S samples, respectively. It is interesting to observe that the surface area of the F110S samples is approximately the double of the other sulphated E400S sample. This difference accounts for the approximate double amount of sulphate groups bonded to the F110S sample in comparison to the E400S one.

### 2.2. Spectroscopic Characterization of the Bare and Sulphated Tin Oxide Powders

All the SnO_2_ samples, both bare and sulphated, have been investigated by vibrational spectroscopies (FTIR and Raman) in order to investigate the modes of the surface groups and the nature of the chemical bond between the sulphates and the SnO_2_ surfaces. 

**Figure 4:**
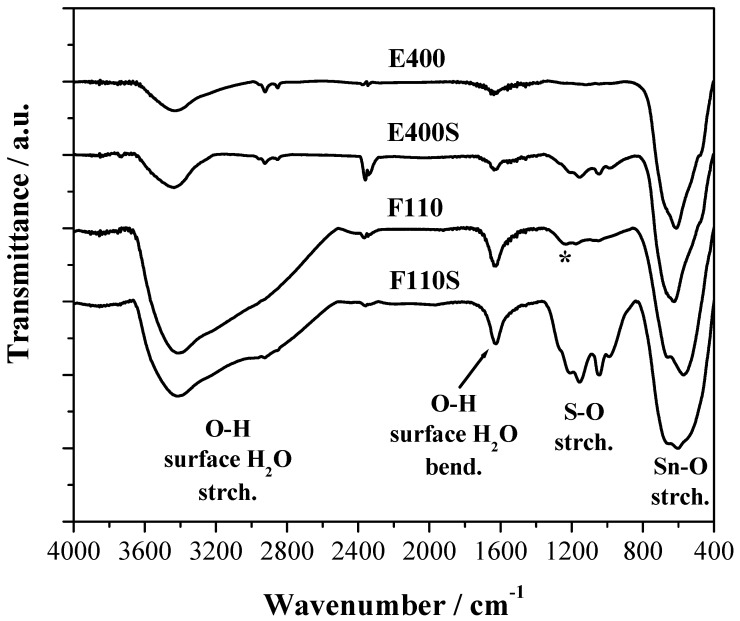
Fourier Transform Infrared (FTIR) spectra of the bare and sulphated oxides. The (*) symbol corresponds to the Sn–OH bending mode.

The FTIR spectra of the four samples are reported in the Figure 4 where three main spectral features are observed. All samples show a broad composite band due to the vibrational modes of the Sn–O–Sn oxide lattice below 800 cm^−1^ and a band at about 1620 cm^−1^ attributed to the bending mode of surface water molecules and –OH groups [34].

The presence of physisorbed water and OH terminal groups on the surface of all the samples, even after calcination at 400 °C and/or 500 °C, is confirmed by the large broad band due to the stretching of the OH bond between 2400 and 3600 cm^−1^. The F110 sample shows in addition a composite response between 1250 and 800 cm^−1^ that is likely due to the bending of Sn–OH bonds as discussed in [35]. 

For the two E400S and F110S sulphated samples a series of FTIR bands between 1400 and 800 cm^−1^ due to the sulphate stretching modes are evident. As expected, their intensity is stronger for the F110S sample that has a double amount of sulphate groups bonded to the surface compared to the E400S sample. It is to be noted that the position and relative intensity of the bunch of narrow bands due to sulphate groups are very similar for the two samples: this evidence suggests that the nature of the chemical bond between the SnO_2_ oxide and the sulphate groups bonded is almost the same in the two samples.

**Figure 5:**
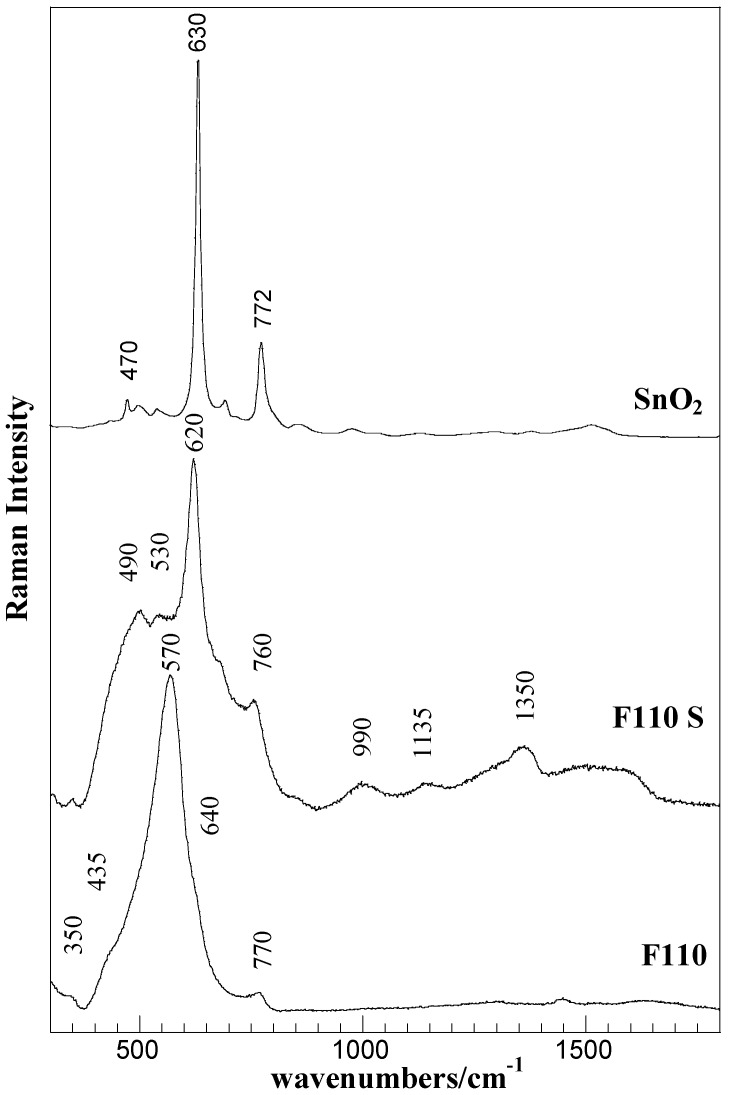
Raman spectra of the F110 and F110S samples and of bulk SnO_2_ (Aldrich).

The evolution of surface vibration modes, revealed by Raman spectroscopy, are presented in Figure 5 for the F110 and F110S samples, as a representative case. The spectrum of bulk SnO_2 _is also shown for comparison.

The Raman spectrum of the starting F110 sample mainly consists of a broadened strong band centered at about 570 cm^−1 ^and of low intensity bands at about 350 cm^−1^, 435 cm^−1^, 640 cm^−1^, and 770 cm^−1^. According to factor group analysis, four first-order Raman active modes (A_1g_, B_1g_, B_2g_, E_g_) are predicted for crystalline SnO_2_ (tetragonal rutile structure) at 100 cm^−1^ (B_1g_), 479 cm^−1^ (E_g_), 638 cm^−1^ (A_1g_), and 779 cm^−1^ (B_2g_) [36]. The bands at about 570 and 350 cm^−1^ are attributed to interface or surface phonon modes of nanostructured SnO_2_ material [37,38], being this in agreement with the results of XRD and morphological analyses. As, at decreasing particle size, a shift to lower wavenumbers is expected, the strong band at about 570 cm^−1^ can be related to the A_1g_ mode of the SnO_2 _structure, whereas the barely detectable bands at about 435 cm^−1^, 640 cm^−1^, and at about 770 cm^−1^ are attributed to normal interior phonon modes of the bulk SnO_2_ structure.

The sulphation process yielded a strong modification in the Raman spectra of the F110 sample. In addition to the changes in the SnO_2_ structure, revealed in the 300–800 cm^−1^ spectral region, the presence of sulphate-species were detected in the range 930–1600 cm^−1^ of the F110S sample. The sharp and intense bands at about 490, 620, and 760 cm^−1^ are ascribed to SnO_2_ in crystalline form (E_g_, A_1g_, and B_2g_ modes) [36], the amorphous nanostructured starting material is, however, still present as evidenced by the large band underlying the whole region representative of surface phonon modes.

The bands in the 900–1500 cm^−1^ range, at about 1000, 1150, and 1360 cm^−1^, disclose the presence of various surface sulfate species. Although bands assignment is still conflicting, the bands at about 1000 and 1350 cm^−1^ can be attributed to S–O and S=O modes of the surface sulfate groups [39]. The band at about 1135 cm^−1 ^can also be attributed to S–O stretching modes, but in polynuclear sulfate groups and/or stretching modes in bidentate sulfate species [40]. Furthermore, the broad band centered at about 1600 cm^−1^ can be assigned to the OH vibration of adsorbed molecular water.

### 2.3. Physico–Chemical Characterization of the Nafion-based Membranes

Five membranes have been prepared without and with the four filler powders. The filler loading has been chosen as 5 wt % in agreement with previous results published by Chen *et al*., where Nafion membranes containing 5 wt % of sulphated tin oxide were shown to have optimized proton conductivity with respect to undoped and 10 wt %-added membranes [30]. Filler amount not higher than 4–5 wt % has been demonstrated to be the most effective also for other metal oxide-doped Nafion membranes, both in terms of homogeneous dispersion of the additive within the polymer matrix and of improved stiffness of the resulting composite system [21,41]. 

A summary of the membrane compositions and the corresponding water uptake (WU) and ionic exchange capacity (IEC) values are reported in the Table 1 together with data derived from the thermal analyses.

**Table 1:** Water uptake (WU), ionic exchange capacity (IEC), and parameters derived from the thermal analysis for the five prepared membranes.

Code	Filler (5 wt %)	IEC mequiv g^−1^	WU	ΔH/J g^−1^ (DSC)	T_tr_/°C (DSC)
N	none	0.880	37%	76.9	185
N-E400	E400	0.834	38%	105	163
N-E400S	E400S	0.812	37%	93.3	153
N-F110	F110	0.827	40%	114	146
N-F110S	F110S	0.820	40%	181	153

The ionic exchange capacity decreases slightly for all the composite membranes compared with the benchmark recast Nafion (N). This is likely ascribed to the fact that the ceramic powder addition is expected to increase the density of the membrane thus reducing the amount of acid sites for mass units. The small differences in IEC values among membranes containing sulphated or unsulphated filler are not considered of relevance. Indeed, due to the nature of the experiment adopted for the IEC determination (see Experimental section), incomplete ion exchange can even be considered, mainly when compounds with very high proton affinity are being used.

**Figure 6:**
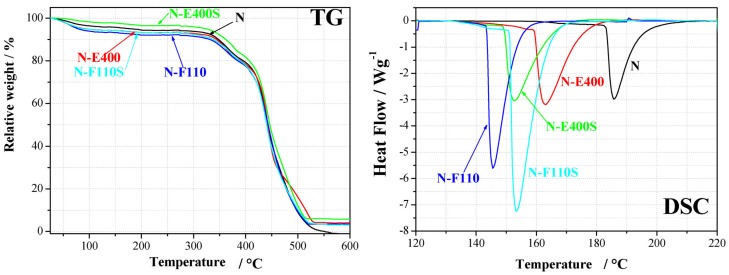
Thermal analysis results (TG and DSC) for the five membranes.

The effect of the fillers on the hydration/dehydration of the membranes is proved by water uptake and thermal analysis results. The WU is apparently slightly higher in the case of the composite membranes containing the F110 additive, both sulphated and non. However these data do not provide information on the nature and the strength of the bonds between water, filler, and Nafion ionic domains. The thermal profiles of the membranes from TG and DSC experiments are shown in Figure 6.

The TG analyses show, as expected, two main mass loss processes: below 200 °C and above 300 °C, respectively. The first process is related to the removal of water from the polymer matrix, whereas the processes above 300 °C to the degradation and combustion of the polymer matrix. The residual mass above 500 °C is due to the ceramic filler; values, slightly differing from the theoretical amount of 5 wt %, are ascribed to small non-homogeneity within the samples. The composite membranes added with the sulphated oxide fillers, *i.e.*, N-E400S and N-F110S, show a mass loss around 120 °C slightly smaller than those observed for membranes added with the bare oxides, N-E400 and N-F110, respectively. The membrane hydration above 100 °C is a key technological property as such a temperature range is a practical target for PEM fuel cell operation. This experimental evidence may suggest a content of water smaller for the N-E400S and N-F110S samples than for the N-E400 and N-F110 parent membranes. On the other hand, one cannot exclude the presence of highly coordinated or bonded water, strongly interacting with the fillers, not easy to be removed at the quoted temperature. Owing to this and to the nature of TGA experiments (see Experimental section), where membrane samples are not equilibrated under stable temperature and/or relative humidity states, the mass losses at 120 °C can not be taken as a metric for evaluating water content in the membranes.

DSC experiments show for all samples the expected broad endothermic peaks between 140 and 190 °C. This thermal effect is due to an order-disorder transition in the ionic domains of the Nafion polymer membrane [42]. It is known to be strongly affected by the amount of water within the membrane both in terms of transition temperature (T_tr_, smaller for larger water contents) and enthalpy variation (ΔH, larger for higher hydration degrees) [21]. All the membranes added with the inorganic fillers show smaller transition temperatures and larger enthalpy variations in comparison with the Nafion benchmark membrane. Thus, the addition of SnO_2_-based inorganic fillers univocally leads to an increase of the amount of water within the composite membranes under the experimental conditions here quoted (*i.e.*, without any control of external relative humidity). In particular the comparison among the DSC data for the N-E400, the N-F110 and the benchmark N membrane suggests that the N-F110 sample keeps the larger amount of water. This is in agreement with the TG results (see Figure 2) and with the WU measurements (see data in Table 1).

Focusing the attention to the sulphated membranes, the DSC data show contradictory trends. In particular, the N-E400S sample shows a decrease of the transition temperature and of the enthalpy variation compared to the bare N-E400 membrane, whereas the N-F110S sample shows an increase of the transition temperature and of the enthalpy variation compared to the bare N-F110 membrane (see data in Table 1).

In the attempt to understand this behavior, relative interactions among components on a molecular level and the water affinity of the four oxide fillers have to be considered. As shown by the TG data reported in Figure 2, the mass loss due to water desorption from the oxides is different in the four cases. In particular, the E400 and the E400S oxides show a similar mass loss <1% up to 160 °C, whereas the F110 and the F110S samples suffer larger losses, *i.e.*, 6% and 4%, respectively. Owing to this, it is likely that also the amount of water in the N-E400 and N-E400S is quite similar. In this view it is possible to speculate that the H_2_O molecules are more strongly bonded in the N-E400S membrane compared to the N-E400 sample and that the addition of the sulphated E400S filler enhances the retention of water above room temperature. This is in agreement with the decrease of the order-disorder transition temperature observed in the DSC data between N-E400 and N-E400S. The parallel contradictory smaller enthalpy variation may be related to the larger strength of the chemical bond between water molecules and the oxides particles in the case of the N-E400S membrane, this reducing the expected effect of water towards the polymer ionic clusters in terms of cohesive interactions. In fact, the sulphate groups on SnO_2_ may strongly coordinate the residual water around the inorganic fillers particles, thus, altering the mean amount of free water dispersed throughout the Nafion matrix.

Turning back to the F110 and F110S samples, the TG data reported in Figure 2 suggest larger mass losses for the F110 oxide in comparison to F110S, excluding in the estimate the loss due to sulphate group removal. In this view it is likely that the amount of water in the composite matrix is much larger for the N-F110 membrane compared to the N-F110S. This is in agreement with the lower order-disorder transition temperature observed in the DSC for the N-F110 sample compared to the N-F110S membrane. Here, the higher enthalpy variation observed for the N-F110S sample in comparison with the N-F110 one may be related to enhanced interactions between water molecules and ionic clusters, this suggesting the existence of differently coordinated and interacting water in the various membranes. In summary the addition of the SnO_2_-based inorganic filler promotes hydration into the Nafion-based membranes, as shown by both WU measurements and thermal analyses. Furthermore the surface modification of the filler, due to the addition of sulphate groups, apparently modifies the strength and the nature of the water-filler and water-ionic cluster bonds. 

### 2.4. Atomic Force Microscopy (AFM) and Dynamic Mechanical Characterization of the Composite Membranes

The morphologies of the composite membranes, as well as of the benchmark Nafion, all in their dried state (*i.e.*, 1 h treatment at 70 °C in air), were investigated by AFM in tapping mode. 

**Figure 7:**
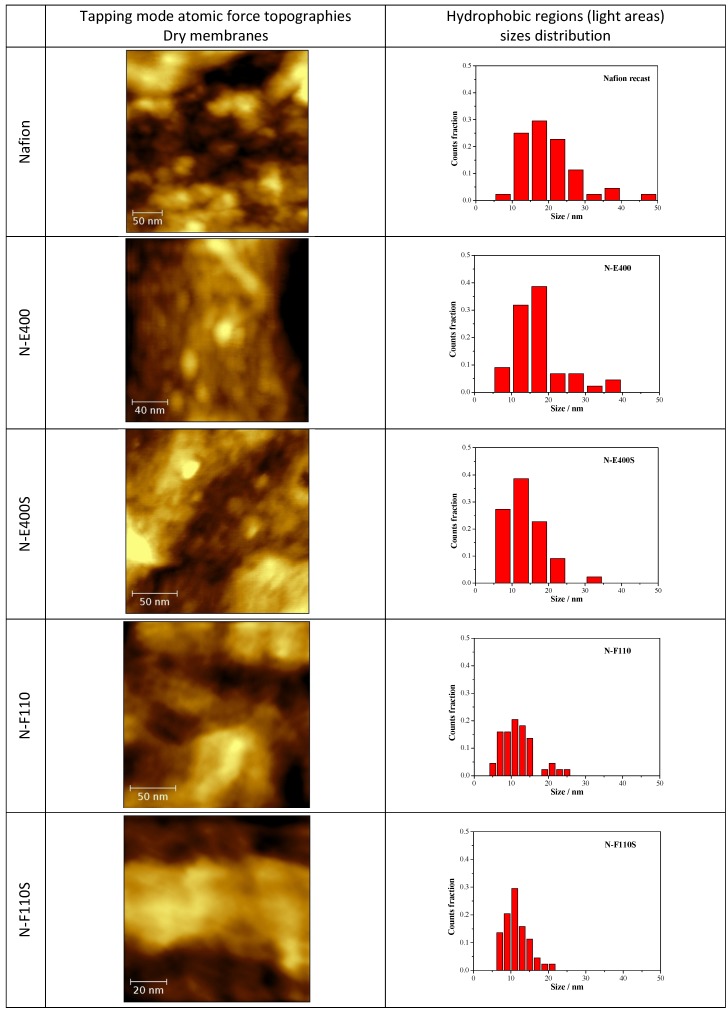
Atomic force microscopy (AFM) topographies measured in tapping mode of the five dried membranes and size distribution of the hydrophobic regions as derived from the image analysis.

In Figure 7 the tapping mode AFM topographies of the five membranes in their dried state are compared together with the size distribution of the round-shaped pseudo-particles (see below) derived from the image analysis by the software ImageJ [43] of five different AFM micrographies at different magnifications for each sample. AFM experiments have been carried out on both sides of the membranes without observing remarkable differences.

The N, N-E400, and N-E400S dried membranes show quite similar surfaces with round shaped aggregated pseudo-particles of different sizes (light regions). This morphology (*i.e.*, agglomerated round shaped pseudo-particles separated by darker areas) can be ascribed to the alternation of the hydrophobic (light regions)/hydrophilic (dark regions) domains across the polymer matrix due to the interaction of the sulphonic groups dispersed within the hydrophobic network of the Nafion polymeric backbone [44,45,46,47]. The dimension (<d>, mean diameter of the hydrophobic regions (light round-shaped pseudo-particles) is much smaller and more homogenously dispersed in the case of the two membranes added with E400 (<d> = 16.6 ± 6.7 nm) and E400S oxides (<d> = 13.9 ± 5.4 nm) in comparison with the benchmark N (<d> = 20.0 ± 8.9 nm). Furthermore, the hydrophilic domains (dark areas) surrounding the pseudo-particles increase their size and morphological regularity passing from the recast Nafion membrane to the N-E400 and the N-E400S membranes. Apparently, the addition of the E400 and E400S oxides leads to a more homogeneous dispersion of the hydrophilic/hydrophobic domains and to a reduction of the size of the latter. The size of the light areas matches those observed for similar samples in the literature (e.g., [47]) where cluster-like structures with diameter of 5–30 nm are typically observed. Furthermore, superstructure of spherical domains with an average diameter of tenths of nanometers containing 10–12 nm grains are usually detailed in AFM images [47]. Among these grains, dark interstitial regions of few nanometers are observed being the latter an estimate of the ionic cluster size. Apparently, our observation matches this picture.

The alterations of the morphology of the three samples are induced by the oxide particles. Although these changes may be easily put into correlation with the possible larger hydration of the hydrophilic domains, they may result from two different and interacting phenomena difficult to decouple, *i.e.*, (a) the alteration of the re-casting mechanism during membrane preparation (see experimental part) that reflects on the local organization of the ionic/hydrophobic domains; (b) the possible larger water retention in the membrane after drying due to the stronger bonding of the water molecules mediated by the oxide surfaces. 

On passing one may observe that the size of the oxide particles (both E400 and E400S, see XRD and morphological analyses results) matches that of the round shaped ionic domains. Unfortunately our AFM investigation did not give clear evidence of filler particles: in our experience, it is difficult to distinguish between the ionic domain and the similar-in-size filler particles without referring to the AFM phase-images, where the harder oxide particles may give evidences. As a consequence, the here reported AFM study did not provide any undoubted evidence of the presence of filler particles embedded in the polymer matrix. In this view a further interpretation of the possible origin of the larger hydration of the ionic channels based on our AFM data would be too speculative.

A larger surface morphology alteration is observed for both the N-F110 and N-F110S. In particular, although as expected the overall morphologies of the N-F110 and the N-F110S samples still resemble those of the benchmark Nafion and the N-E400 and N-E400S membranes, the surfaces are apparently smoother with less resolved alternation of the hydrophilic/hydrophobic domains. The size-distribution plots suggest a mean size of the hydrophobic pseudo-particles of about 12.0 ± 4.4 and 11.2 ± 3.2 nm for the N-F110 and the N-F110S samples, respectively. Moreover also the hydrophilic regions surrounding the light hydrophobic domains show round-shaped morphologies and sizes matching those of the light areas. In addition, in the cases of the N-F110 and the N-F110S membranes the modification of the morphologies may be ascribed to the increase of the hydration of the ionic channels resulting from the alteration of the re-cast process and/or to the larger water retention after drying. Moreover it is interesting to underline that, differently from the cases of the N-E400 and N-E400S membranes, in both the N-F110 and N-F110S cases the particle size of the inorganic fillers estimated from surface area measurements is much smaller in comparison with the size of the round-shaped pseudo-particles and the surrounding darker areas. One may speculate that, in these two cases (N-F110 and N-F110S), the oxide particles may float well within the hydrophilic channels thus improving the water retention without inducing a large mechanical stress on the surrounding polymer matrix.

**Figure 8:**
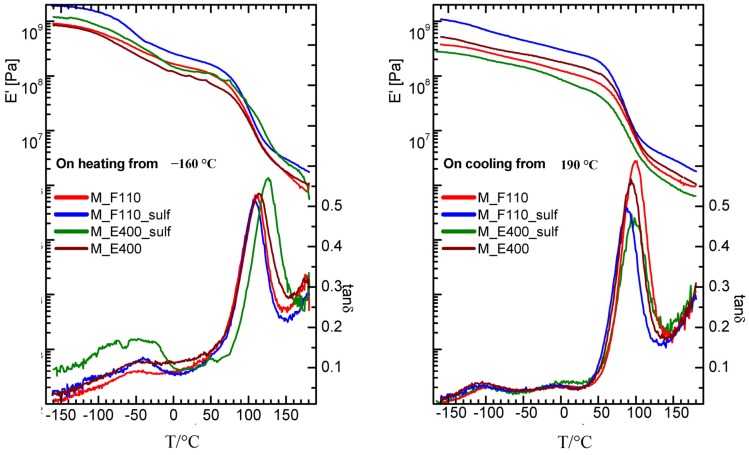
Storage modulus and elastic energy dissipation of nanocomposite membranes measured for f = 1 Hz on heating (left panel) and subsequent cooling (right panel).

Turning to the mechanical properties, the storage modulus (E’) and elastic energy dissipation (tanδ) of the nanocomposite membranes (N-E400, N-E400S, N-F110, and N-F110S), measured starting from a “wet membrane” state on heating and subsequent cooling, are reported in Figure 8.

On heating starting from −160 °C, the α relaxation (the intense peak in tanδ and the two order of magnitude drop in the modulus) is detected around 110 °C in the F110-added (either sulphated or not) and in the E400-added nanocomposite membranes, while it results slightly shifted to higher temperature (120 °C) in the sulphated E400-added sample. This relaxation corresponds to the glass transition of the hydrophilic domains (polar regions) of the Nafion polymer matrix. On cooling, the peak associated to the alpha relaxation is measured at about 80 °C (see right panel of the figure) in all membranes, showing thermal hysteresis. Moreover, on heating between −90 and 30 °C, all the nanocomposite membranes show a broad peak, accompanied by an increase in the modulus. This feature has been already observed in a wet undoped Nafion membrane [48] and attributed to the friction between the liquid water bound to the walls of the hydrophilic domains and the solid ice inside the channels. The intensity of this peak is higher in the membrane with the sulphated oxide as filler, and is maximum in the sulphated samples prepared by the E400 procedure (N-E400S). It can be speculated that the higher intensity of the broad peak below 30 °C in N-E400S is related to the distribution and to the particular status of water molecules within the composite matrix. Even though a sort of preferential interactions between water and this particular filler (E400S) in Nafion membrane has been also revealed by DSC measurements (see discussion above), further insights are needed to better explain this evidence, thus complementary investigations are currently in progress. 

The effect of hydration on the properties of ionomers and polyelectrolytes is, in general, a very complex, still open question. It is known that many variables, such as external relative humidity, temperature, and, as recently demonstrated by Kreuer, internal pressure contribute to define the hydration thermodynamics and the transport properties across the membrane [49]. When inorganic nanoparticles are introduced into a polymer electrolyte, the picture becomes even more complex and hydration is altered [50].

Our findings show that the storage modulus and tanδ measured for all the nanocomposite membranes on cooling immediately after heating at 180 °C are close and similar to those already reported for a dry, undoped Nafion membrane [48] as they do not show anymore neither the broad peak nor the corresponding increase in the modulus due to the reinforcing action of solid water. Moreover, when measured on cooling, the gamma and beta relaxations are more evident around −70 and 0 °C, respectively, as the corresponding peaks are more separated. The beta relaxation has been ascribed in the literature to the glass transition of the Nafion non-ionic matrix, while the gamma relaxation was assigned to short-range molecular motions of the –CF_2_– backbone. 

There are some interesting examples in literature where the viscoelastic properties of Nafion have been studied as a function of the hydration level and of temperature and correlated to the transport properties across the polymer membrane [49,51]. In the present work, the effect of the particles addiction on the mechanical properties of the membranes can be discussed by the measurements of the storage modulus in the cooling run (right panel of Figure 8), where most of the water may have evaporated. Most of the composite samples present values higher than that of pure recast Nafion (results not shown here) and, in particular the N-F110S sample displays the highest modulus, suggesting a reinforcing action due to the incorporation of the F110S sulphated tin oxide particles. An increase of the Young modulus in a Nafion membrane with the addition of SnO_2_ nanoparticles was already observed by Nørgaard *et al*. [41] and related to a stiffening effect due to interfacial interactions between the inorganic phase and sulfonic acid groups in the polymer. 

The applicability of the proposed composite membranes as effective electrolytes needs, of course, to be demonstrated. To this purpose, a detailed characterization, including electrochemical measurements of proton conductivity and fuel cell operation, will be reported in another publication aimed at elucidating the practical impact of the selected electrolytes under critical conditions [52]. It’s here worth mentioning that interesting conductivity values were obtained for the two sulfated tin oxide-added membranes, ranging from 7 × 10^−3^ to 9 × 10^−3^ S cm^−1 ^at room temperature under 100% RH.

## 3. Experimental Section

### 3.1. Preparation of SnO_2_-Based Inorganic Powders

Highly hydrated nanosized SnO_2_ powders were obtained by two different methods:

1. Sol-gel synthesis from Sn(II)-2-ethyl-hexanoate [31]. 5 mL of the organic Sn-precursor (Sigma Aldrich, 97%) were added dropwise to 50 mL of n-propanol. A volume of 1 mL of concentrated NH_3_ (30% weight) was added dropwise to the Sn(II) alcoholic solution under vigorous stirring. The yellowish precipitate was separated by centrifugation and then calcined at 400 °C in air. The obtained material was denoted as E400.
2. Aqueous hydrolysis of SnCl_4_ [32]. SnCl_4_ (Fluka, 99%) was dissolved in water (2 M solution) and then Sn(OH)_4_ was precipitated by adding dropwise to 20 mL of the Sn(IV) solution 10 mL of concentrated (30% weight) water solution of NH_3_. The resulting precipitate was carefully washed with de-ionized water to remove the NH_4_^+^ and Cl^−^ residual contaminations. The final white gel was dried under dynamic vacuum overnight at 110 °C and then hand grinded. The sample was designated as F110.

The sulphated tin-oxides (named E400S and F110S) were obtained by chemical treatment followed by calcinations of the E400 and F110 samples. In both cases the starting oxide was suspended in de-ionized water and stirred continuously in order to enhance the water adsorption. After 24 h of vigorous stirring the water suspension was treated with H_2_SO_4_ (final concentration 0.5 M) for 30 min under stirring. After decantation, the material was filtered and then calcined in air for 3 h at 500 °C.

In summary four inorganic fillers were prepared:

- E400 and E400S from alcoholic hydrolysis of Sn(II)-2-ethyl-hexanoate;
- F110 and F110S from the water hydrolysis of SnCl_4_.

### 3.2. Preparation of the Composite Membranes

Nafion membranes, both without and with the inorganic filler, were prepared following a solution casting procedure. The commercial Nafion dispersion (5 wt % in water/alcohol, E.W. 1100, Ion Power, GmbH, Munchen, Germany) was treated with *N*,*N*-dimethylacetamide at 80 °C in order to replace solvents. When required, inorganic filler powders were added to the final Nafion solution and stirred to homogenize the suspension. An optimal additive loading of 5% with respect to the Nafion polymer weight was chosen for our investigations [30]. The mixture was casted on a Petri dish and dry membranes were obtained by solvent evaporation at 90 °C. The casted polymer membranes, having a geometrical surface area of *ca.* 28 cm^2^, were hot-pressed at 175 °C for 15 min at 10 tons to improve robustness and finally activated and purified by subsequent immersions in boiling hydrogen peroxide (3 vol %), sulfuric acid (0.5 M), and water. All the prepared membranes showed very similar thicknesses of about 90 µm.

Membranes containing the inorganic fillers are labeled in the text as N-E400, N-E400S, N-F110, and N-F110S, respectively. Undoped membranes, used as internal benchmark, will be referred to as recast Nafion and labeled as N.

### 3.3. Experimental Methods

The XRD patterns on the synthesized inorganic ceramic powders were recorded with a step size of 0.02° and a time per step of 3 s, with a Rigaku Ultima+ diffractometer equipped with a CuKα radiation source and a graphite monochromator in the 2θ range of 15°–85°. 

The morphology of powders and membranes was studied by the SEM Phenom FEI with a LaB_6_ source and by the Park AFM XE120 instrument in tapping intermittent contact mode (TM), respectively. It is important to underline that phase-distance curves were carefully monitored upon imaging in order to avoid the occurring of unstable regimes [45] and the rise of height artifacts in AFM topographies.

Single point N_2_-adsorption experiments for surface area determination were carried out by the Quantachrome Monosorb instrument. All measurements were performed in triplicate on two different sample batches for each material (F110, F110S, E400, and E400S). Each sample was carefully pre-treated before N_2_ adsorption at 120 °C for 9 h in order to remove physisorbed moisture.

FTIR spectroscopy studies were performed by using a Bruker Alpha FTIR apparatus in transmission mode. 

Raman spectra were collected at room temperature in back-scattering geometry with an inVia Renishaw micro-Raman spectrometer equipped with an air-cooled CCD detector and super-Notch filters. A 488.3 nm emission line from an Ar^+^ ion laser was focused on the sample by a Leica DLML microscope, using 5× or 20× objectives. The power of the incident beam is about 5 mW. Five 10 s accumulations were generally acquired for each sample. The resolution was 2 cm^−1^ and spectra were calibrated using the 520.5 cm^−1^ line of a silicon wafer. 

Thermal analyses were carried out by differential scanning calorimetry (DSC) using a DSC 821 Mettler-Toledo instrument, and by thermal gravimetric (TG) analysis performed with a TGA/SDTA851 Mettler-Toledo instrument. DSC measurements were realized on membrane samples at a scan rate of 20 °C min^−1^ in a nitrogen flux. TG analysis was executed in air flux on both tin oxide particles and membranes, at a heating rate of 5 °C min^−1^ and 20 °C min^−1^, respectively. Both, TG and DSC analyses of polymer membranes, composite and bare, were performed on membrane samples stored in water, by simply removing surface water drops with the help of technical paper wipes prior to measurements.

Dynamic mechanical analysis was realized by a Perkin-Elmer DMA8000 apparatus in the so-called “tension configuration”. Small pieces 4–7 mm wide, 10–12 mm long, 0.10–0.15 mm thick were cut from the various membranes stored in water. The storage modulus (E’) and the elastic energy loss (tanδ) were measured, with a vibration frequency f = 1 Hz, as a function of temperature in the range of -160–190 °C with a scan rate of 4 °C/min. 

Membrane total water uptake (WU) was measured at room temperature. Samples were dried at 70 °C under vacuum overnight and then weighted (*w*_dry_). The weight of fully hydrated membranes (*w*_swollen_) was obtained immediately after the treatment in boiling water at the end of the activation procedure. The WU value was evaluated by the formula

WU = (*w*_swollen_ − *w*_dry_)/(*w*_dry_) × 100%

The ion-exchange capacity (IEC), which provides the number of milli-equivalents of ions in 1 g of dry membrane, was evaluated by a classical titration method: dry membrane samples were immersed in NaCl aqueous solution and the exchanged protons were neutralized with OH^−^.

## 4. Conclusions

The properties of nanocomposite ceramic-added polymer membranes were investigated by means of complementary physical chemical characterization tools. The synthesis and the investigation of highly hydrophilic nanosized tin oxide fillers, without and with sulphated surfaces, and their incorporation into a selected polymer matrix have been described. Two nanometric SnO_2_ materials, differing in crystallite size and surface area, have been successfully prepared starting from different precursors. Both materials have been sulphated by a tailored procedure in order to obtain superacidic SnO_2_ nanoparticles. All the inorganic fillers have been incorporated into Nafion matrices to obtain the desired nanocomposite, hybrid membranes. It was demonstrated that both the particle dimension and the sulphation extent of the additive determined a different hydration degree, this in turn affecting the final properties of the doped membranes. Even though this work was not specifically aimed at elucidating the hydration concern within composite Nafion systems some interesting evidences were found when comparing samples of slightly different nature under the same experimental conditions here quoted. Indeed, calorimetric, dynamic-mechanical and atomic force investigations suggested the occurrence of distinctive interactions among the various components (*i.e.*, ceramic, polymer, and absorbed water), being these relative interactions strongly dependent on the type of SnO_2_ considered. In particular, smaller crystallite size and higher surface sulphation of the ceramic particles seemed to promote a larger water affinity, a more regular organization of the polymer ionic channels and an increase in the storage modulus of the composite membrane. 

All these findings are highly encouraging for possible applications of the systems here proposed as advanced electrolytes in proton exchange membrane fuel cells, mainly in view of the targeted requirement concerning applications under low relative humidity. 
